# Supplementary material for: The Construction and Exploration of a Comprehensive MicroRNA Centered Regulatory Network in Foxtail Millet (Setaria italica L.)
Source: Front Plant Sci. 2022 May 6;13:848474. doi: 10.3389/fpls.2022.848474 (PMC9121102; doi:10.3389/fpls.2022.848474)
Supplement: Supplementary file 2 [file Data_Sheet_2.zip › Sit-miR396c_Seita.3G361100.1_2996_TPlot.pdf]

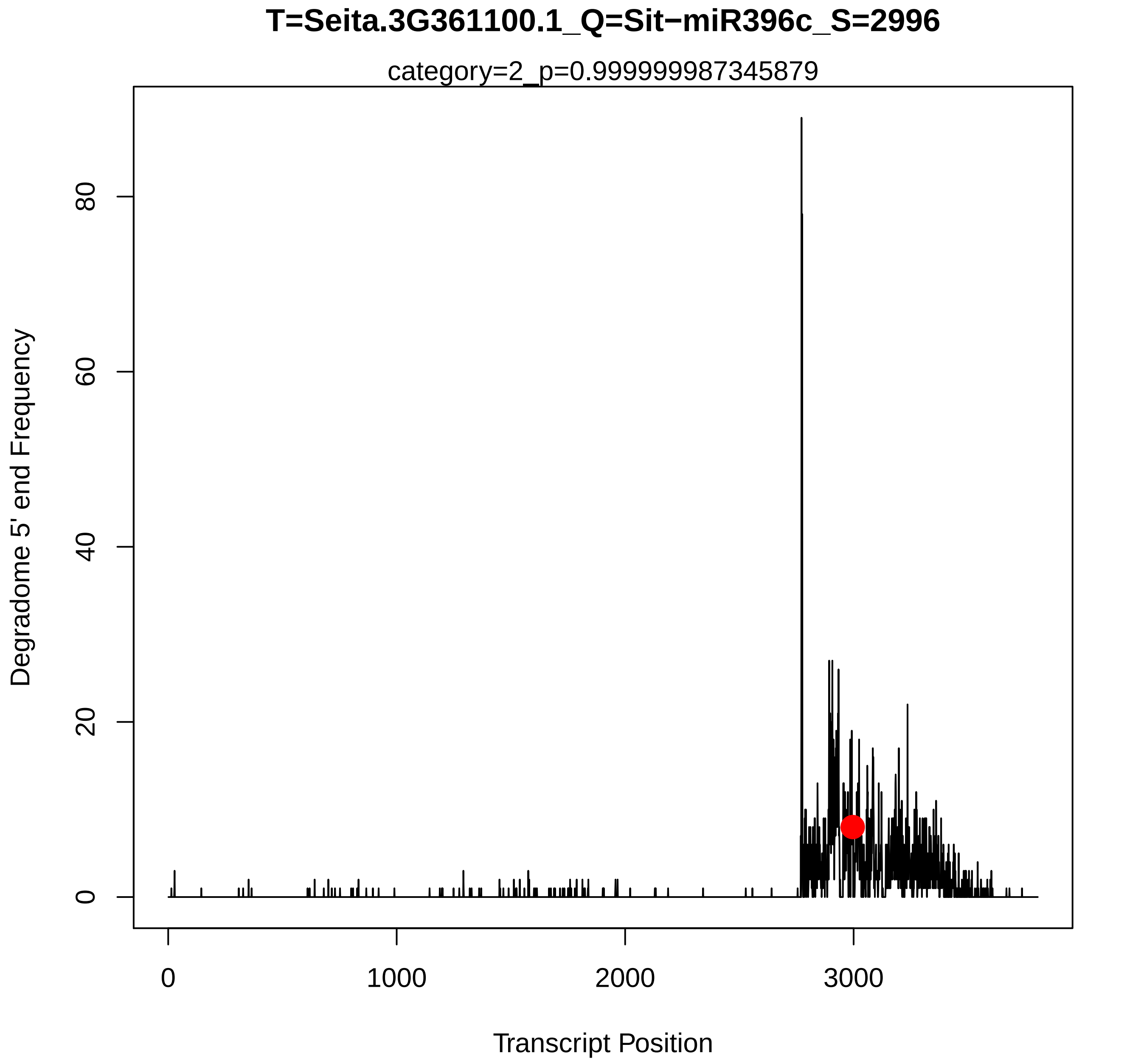
T=Seita.3G361100.1_Q=Sit−miR396c_S=2996
category=2_p=0.999999987345879
Degradome 5' end Frequency
0
20
40
60
80
0
1000
2000
3000
Transcript Position
